# How many replicates to accurately estimate fish biodiversity using environmental DNA on coral reefs?

**DOI:** 10.1002/ece3.8150

**Published:** 2021-10-06

**Authors:** Salomé Stauffer, Meret Jucker, Thomas Keggin, Virginie Marques, Marco Andrello, Sandra Bessudo, Marie‐Charlotte Cheutin, Giomar Helena Borrero‐Pérez, Eilísh Richards, Tony Dejean, Régis Hocdé, Jean‐Baptiste Juhel, Felipe Ladino, Tom B. Letessier, Nicolas Loiseau, Eva Maire, David Mouillot, Maria Mutis Martinezguerra, Stéphanie Manel, Andrea Polanco Fernández, Alice Valentini, Laure Velez, Camille Albouy, Loïc Pellissier, Conor Waldock

**Affiliations:** ^1^ Landscape Ecology Institute of Terrestrial Ecosystems Department of Environmental Systems Science ETH Zürich Zürich Switzerland; ^2^ Unit of Land Change Science Swiss Federal Research Institute WSL Birmensdorf Switzerland; ^3^ MARBEC Univ. Montpellier CNRS IFREMER IRD Montpellier France; ^4^ CEFE Univ. Montpellier CNRS EPHE‐PSL University IRD Univ. Paul Valéry Montpellier 3 Montpellier France; ^5^ Institute for the Study of Anthropic Impacts and Sustainability in the Marine Environment National Research Council Rome Italy; ^6^ Fundación Malpelo y otros ecosistemas marinos Bogotá Colombia; ^7^ Instituto de Investigaciones Marinas y Costeras‐INVEMAR Museo de Historia Natural Marina de Colombia (MHNMC) Santa Marta Colombia; ^8^ SPYGEN Le Bourget‐du‐Lac France; ^9^ Institute of Zoology Zoological Society of London London UK; ^10^ Marine Futures Lab University of Western Australia Crawley WA Australia; ^11^ Lancaster Environment Centre Lancaster University Lancaster UK; ^12^ IFREMER unité Écologie et Modèles pour l’Halieutique Nantes France

**Keywords:** biomonitoring, coral reef diversity, environmental DNA, MOTU, sampling variability, tropical marine ecosystems

## Abstract

Quantifying fish species diversity in rich tropical marine environments remains challenging. Environmental DNA (eDNA) metabarcoding is a promising tool to face this challenge through the filtering, amplification, and sequencing of DNA traces from water samples. However, because eDNA concentration is low in marine environments, the reliability of eDNA to detect species diversity can be limited. Using an eDNA metabarcoding approach to identify fish Molecular Taxonomic Units (MOTUs) with a single 12S marker, we aimed to assess how the number of sampling replicates and filtered water volume affect biodiversity estimates. We used a paired sampling design of 30 L per replicate on 68 reef transects from 8 sites in 3 tropical regions. We quantified local and regional sampling variability by comparing MOTU richness, compositional turnover, and compositional nestedness. We found strong turnover of MOTUs between replicated pairs of samples undertaken in the same location, time, and conditions. Paired samples contained non‐overlapping assemblages rather than subsets of one another. As a result, non‐saturated localized diversity accumulation curves suggest that even 6 replicates (180 L) in the same location can underestimate local diversity (for an area <1 km). However, sampling regional diversity using ~25 replicates in variable locations (often covering 10 s of km) often saturated biodiversity accumulation curves. Our results demonstrate variability of diversity estimates possibly arising from heterogeneous distribution of eDNA in seawater, highly skewed frequencies of eDNA traces per MOTU, in addition to variability in eDNA processing. This high compositional variability has consequences for using eDNA to monitor temporal and spatial biodiversity changes in local assemblages. Avoiding false‐negative detections in future biomonitoring efforts requires increasing replicates or sampled water volume to better inform management of marine biodiversity using eDNA.

## Graphical abstract

Biodiversity estimates from eDNA metabarcoding often uncover only a portion of the diversity in a location. We discover that for species‐rich areas, such as coral reefs, we may need more samples than previously thought to recover and monitor biodiversity in a local area.

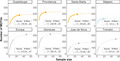

## 1 INTRODUCTION

Biodiversity is changing faster than our ability to accurately quantify species losses and gains (Ceballos et al., 2020; Filgueiras et al., 2021), with consequent difficulties in evaluating the degradation of ecosystem functions and services upon which human well‐being depends (Díaz et al., 2019). Traditional methods such as visual surveys are costly and time‐consuming and require on‐site taxonomic expertise (Ballesteros‐Mejia et al., 2013; Dornelas et al., 2019; Kim & Byrne, 2006). Despite decades of sampling efforts, biodiversity monitoring still covers only a small fraction of global ecosystems and is challenging in isolated and remote regions across the oceans (Collen et al., 2009; Dornelas et al., 2018; Letessier et al., 2019; Webb et al., 2010). An emerging tool for rapid biodiversity assessment is environmental DNA (eDNA) metabarcoding (Eble et al., 2020; Stat et al., 2017), which is proving to be effective in marine environments (Boulanger et al., 2021; Holman et al., 2021; Juhel et al., 2020). eDNA‐based methods rely on the detection of DNA fragments from various sources including feces, shed skin cells, organelles, or extruded waste of animals, which become suspended in seawater (Collins et al., 2018; Dejean et al., 2012; Harrison et al., 2019). Using filtered water and molecular analyses, eDNA metabarcoding can be used to estimate biodiversity across different taxonomic levels without isolating any target organisms (Holman et al., 2021; Valentini et al., 2016) and often without exhaustive genetic reference databases (Flynn et al., 2015; Juhel et al., 2020; Marques et al., 2020, 2021). eDNA metabarcoding overcomes limitations of common sampling methods by targeting complete species assemblages, detecting rare (Rees et al., 2014), elusive (Boussarie et al., 2018), or non‐indigenous species (Ficetola et al., 2008; Holman et al., 2019) and is harmless to organisms (Bohmann et al., 2014; Smart et al., 2016).

Widespread application of eDNA metabarcoding in marine ecosystems faces multiple challenges (Hansen et al., 2018). Importantly, variability sources exist in the recovered biodiversity estimates which are poorly understood (Bessey et al., 2020; Juhel et al., 2020; Rourke et al., 2021; Thalinger et al., 2021). Detection rates and resultant variability in biodiversity estimates depend on eDNA (a) origin (source of an organism's genetic material shed into its environment), (b) state (forms of eDNA), (c) transport (e.g., through diffusion, flocculation or settling, currents or biological transport which can vary according to the depth), and (d) fate (how eDNA degrades and decays) (Barnes & Turner, 2016; Harrison et al., 2019; Thalinger et al., 2021) with DNA particles best preserved in cold and alkaline waters with low exposure to solar radiation (Moyer et al., 2014; Pilliod et al., 2014; Strickler et al., 2015; but see Mächler et al., 2018). As a result, marine eDNA residence time is shorter than in freshwater and ranges from a few hours to a few days (Collins et al., 2018). Marine systems are open, with eDNA particles dispersed by oceanographic dynamics at local (e.g., tides, currents, and water stratification), regional (e.g., eddies), and large (e.g., thermohaline currents) scales. As such, significant dispersal of eDNA from its source may theoretically occur (Andruszkiewicz et al., 2019; Eble et al., 2020); however, many studies indicate that eDNA detection is limited to a small spatiotemporal sampling window (Boulanger et al., 2021; Jeunen, Knapp, Spencer, Lamare, et al., 2019; O’Donnell et al., 2017; Port et al., 2016; Stat et al., 2019; West et al., 2020; Yamamoto et al., 2017). We test whether eDNA sampling strategies need to overcome this potentially high noise‐to‐signal ratio or if small spatiotemporal sampling windows exist that provide a consistent view of local biodiversity.

The most common approach for concentrating marine eDNA is water filtration along transects (Kumar et al., 2020), but the appropriate amount of water to filter remains underdetermined (e.g., 1 L in Nguyen et al., 2020 and 30 L in Polanco Fernández et al., 2020). An increased volume of water should lead to increased compositional similarly among replicates, but even at 2 L 30%–50% of the total species pool were missing in any given sample (Bessey et al., 2020). The question remains whether a larger water volume, which integrates eDNA signal over multiple kilometers, can provide a less variable and more consistent estimate of biodiversity.

In addition to the volume of water, a high level of eDNA sampling replication in the field can be required to reduce false negatives (species present but not detected) and improve the accuracy of biodiversity estimates of local sites and regions. For example, 92 × 2 L seawater samples accurately predict (*R* ^2^ = 0.92) the distribution of species richness for different fish families (Juhel et al., 2020). Spatial diversity gradients have been recovered from only 3 × 0.5 L water samples in temperate (Thomsen et al., 2012) and tropical systems (West et al., 2020). However, West et al. (2020) report that more replicates were necessary to avoid false negatives and better sample diversity in a given site (>8). Budget and time limitations constrain the number of sampling replicates available (Ficetola et al., 2015)—which require optimization to take full advantage of eDNA‐based surveys.

Here, we compared biodiversity of replicated eDNA samples in terms of Molecular Operational Taxonomic Units (MOTUs) since genetic reference databases have many gaps for tropical fishes (Marques et al., 2020). We assessed within‐site MOTU richness (α‐diversity) and between‐site MOTU dissimilarity (*β*‐diversity) separating the turnover and nestedness components (Baselga, 2012). We targeted tropical fishes across eight different sites within the Caribbean, Eastern Pacific, and Western Indian Ocean using the same standardized sampling protocol. Over transects 2 km long, we filtered 30 L of water per sample, with paired samples per transect. In addition, we performed a replication experiment in two locations by repeating transects multiple times in a ~24 h period. Although multiple markers can be associated with greater recovery rates (Polanco‐Fernandez et al., 2021), we used a single primer pair due to cost constraints and thus provide assessment for a pragmatic and cost‐efficient sampling regime. Our objectives were to (a) establish the comparability of fish diversity estimates from replicated eDNA samples collected at the same time, in the same location and under similar conditions, (b) identify the number of eDNA replicates required to saturate diversity curves at a given local site for our protocol (e.g., a using single primer), (c) compare the above patterns among three ecologically distinct tropical ocean regions, and (d) examine whether our sampling protocol saturates regional fish biodiversity estimates. Given that we filtered far more water than previous saturation experiments, we may expect higher eDNA detections whereby MOTU richness and composition should be very similar among the paired replicates—providing robust estimates of biodiversity. In this case, the replicate accumulation curve should saturate rapidly and reach an asymptotic maximum suggesting that the maximum potential diversity for a given sampling design is achieved (e.g., filtering, primer, sequencing methodology). In the opposite case, it would indicate that even a high volume of filtration and a large number of replicates would be required to inventory fish biodiversity regionally.

## 2 METHODS

### 2.1 Sampling sites and eDNA sampling protocol

**FIGURE 1:**
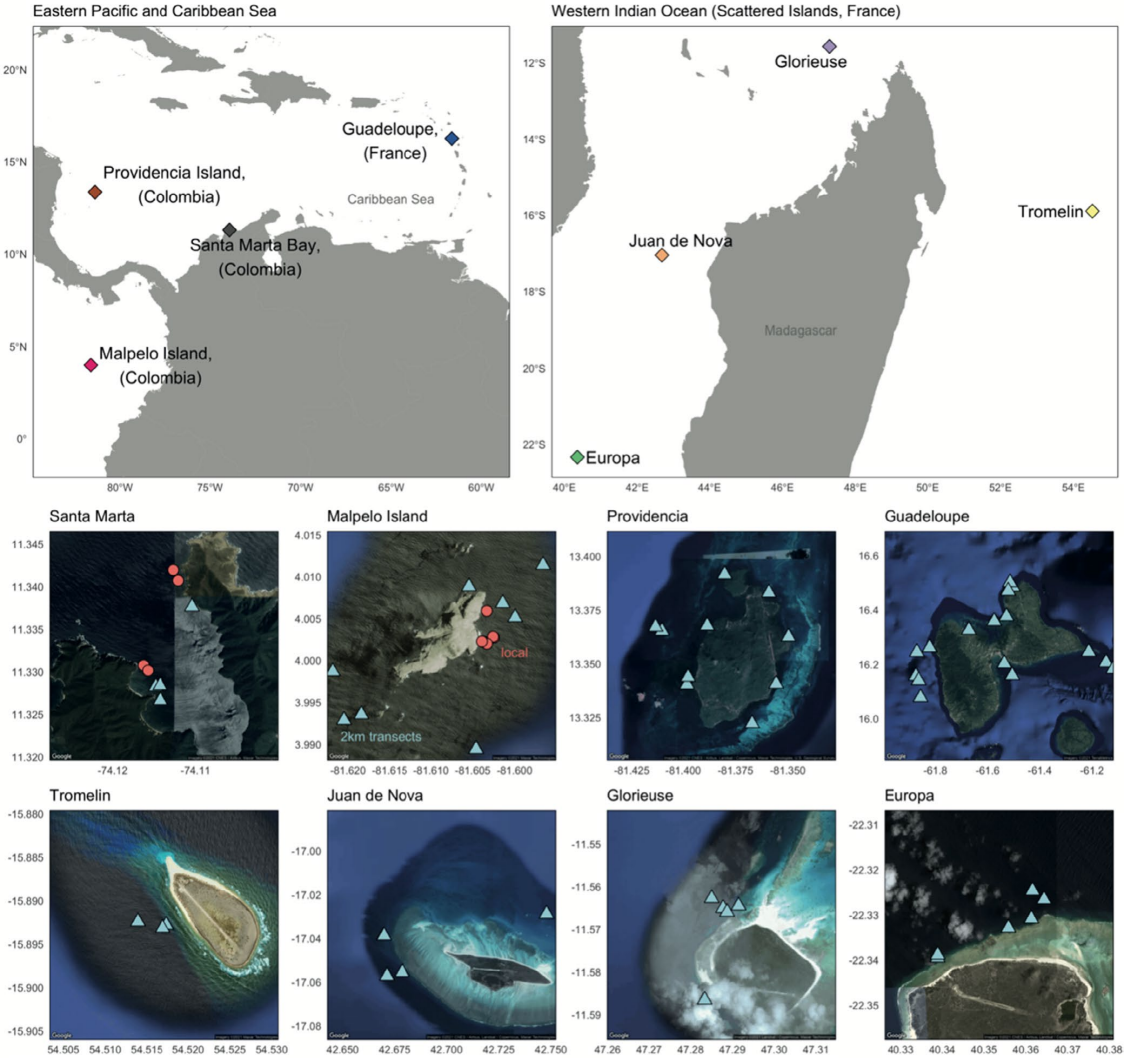
Sampling sites in the Eastern Pacific, Caribbean, and Western Indian Ocean. The eight sampled sites represented by Google Earth imagery show the spatial distribution of transects within sites. Markers represent the beginning of eDNA transects in each site; color and shape indicate whether samples were used in local accumulation analysis (static samples repeated multiple times in a shorter period, red circles) or regional/island level accumulation curves (blue triangles)

We filtered surface seawater across eight sampling sites in three different oceanic regions: Caribbean Sea, Western Indian Ocean, and the Eastern Pacific (Figure 1). At each of the eight sampling sites, several transects were carried out with at least two filtration replicates per transect (see Table 1). Filtration replicates per transect were performed simultaneously on either side of a small boat moving at 2–3 nautical miles per hour while filtering surface seawater for 30 min, resulting in approximately 30 L of water filtered per replicate. The shape of 2 km transect varied to match the configuration of the reefs but were always consistent between the compared replicates. eDNA sampling was performed with a filtration system composed of an Athena^®^ peristaltic pump (Proactive Environmental Products LLC, Bradenton, Florida, USA; nominal flow of 1 L min^−1^), a VigiDNA^®^ 0.2 µM cross flow filtration capsule and disposable sterile tubing for each filtration capsule (SPYGEN, le Bourget du Lac, France). After filtration, the capsules were emptied, filled with 80 ml of CL1 lysis conservation buffer (SPYGEN, le Bourget du Lac, France), and stored at room temperature. A strict contamination protocol in field and laboratory stages was followed using disposable gloves and single‐use filtration equipment. More details can be found in Polanco Fernández et al. (2020).

**TABLE 1:** Overview of eDNA sampling across regions and sites in our study

	Region	Site	Number of transects per site	Total filtration replicates
Local accumulation curves	Eastern Pacific Ocean	Malpelo	5	10
	Caribbean Sea	Santa Marta (#1)	1	6
		Santa Marta (#2)	3	6
Regional accumulation curves	Western Indian Ocean	Europa	6	12 (12)
		Grande Glorieuse	5	10 (10)
		Juan de Nova	5	9 (8)
		Tromelin	3	6 (6)
	Caribbean Sea	Guadeloupe	18	28 (16)
		Providencia	10	20 (20)
	Eastern Pacific Ocean	Malpelo	13	24 (24)
	Caribbean Sea	Santa Marta	8	20 (8)

Note

Filtration replicates are our observation units, all samples in column “*Total filtration replicates”* were used in our accumulation curves. Only paired samples on a given transect were used in sections 3.3 and 3.4, indicated by brackets in the “*Total filtration replicates”* column.

### 2.2 eDNA processing, sequencing, and clustering

eDNA extraction, PCR amplification, and purification prior to library preparation were performed in separate, dedicated rooms following the protocols described in Polanco Fernández et al. (2020) and Valentini et al. (2016). eDNA was amplified using the teleo primer pair (forward: ‐ACACCGCCCGTCACTCT, reverse: ‐CTTCCGGTACACTTACCATG) which targets a ~60 base pair marker within the mitochondrial 12S ribosomal RNA gene and shows high accuracy to detect both bony *(Actinopteri)* and cartilaginous fish *(Chondrichthyes)* (Collins et al., 2019). The primers were 5′‐labeled with an eight‐nucleotide tag unique to each PCR replicate, with forward and reverse tags identical, allowing the assignment of each sequence to the corresponding sample during sequence analysis. Twelve PCR replicates were run per sample, that is, 24 per transect. While sample‐to‐sample variation in PCR replicates exist (O’Donnell et al., 2016), we used a multitube procedure and pooled 12 PCR replicates prior to analyses, which is shown to reduce PCR stochasticity (Tab erlet et al., 1996). Further, this methodology has accurately recovered biodiversity patterns from traditional surveys (e.g., Czeglédi et al., 2021). Fifteen libraries were prepared using the MetaFast protocol (Fasteris). For seven libraries (Caribbean and East Pacific sites), paired‐end sequencing (2 × 125 bp) was carried out using an Illumina HiSeq 2500 sequencer on a HiSeq Rapid Flow Cell v2 using the HiSeq Rapid SBS Kit v2 (Illumina), and for the remaining eight libraries (Western Indian Ocean sites), the paired‐end sequencing was carried out using a MiSeq (2 × 125 bp) with the MiSeq Flow Cell Kit v3 (Illumina), following the manufacturer's instructions. To control for any potential biases linked to the differences in sequencing platforms, the samples were titrated before library preparation to achieve a theoretical sequencing depth of 1,000,000 per sample in each library and sequencing platform. Library preparation and sequencing were performed at Fasteris facilities. Fifteen negative extraction controls and six negative PCR controls (ultrapure water, 12 replicates per PCR control) were amplified per primer pair and sequenced in parallel to the samples to monitor possible contaminants.

To provide accurate diversity estimation in the absence of a complete genetic reference database (Marques et al., 2021), we used sequence clustering and stringent cleaning thresholds (Marques et al., 2020). This procedure has been validated in Marques et al. (2020) and generates highly correlated alpha, beta, and gamma diversity between traditional taxonomic and MOTU‐based diversity estimates (correlation *r* ~ 0.98). Clustering was performed using the SWARM algorithm which uses sequence similarity and abundance patterns to cluster multiple variants of sequences into MOTUs (Fisher et al., 2015; Rognes et al., 2016). First, sequences were merged using *vsearch* (Rognes et al., 2016), next we used *cutadapt* (Martin, 2011) for demultiplexing and primer trimming and finally *vsearch* to remove sequences containing ambiguities. SWARM was run with a minimum distance of one mismatch to make clusters (Marques et al., 2020). Once the MOTUs are generated, the most abundant sequence within each cluster was used as a representative sequence for taxonomic assignment (see Polanco Fernández et al., 2020 for details). We applied a postclustering curation algorithm (LULU) to identify potential errors, using sequence similarity and co‐occurrence patterns, which curates the data by removing MOTUs identified as artifactual without discarding rare but real MOTUs (Frøslev et al., 2017). We removed all occurrences with less than 10 reads per PCR. Finally, we removed all MOTUs present in only one PCR replicate within the entire data set. This additional step was necessary as PCR errors were unlikely to be present in more than one PCR occurrence, and it removed spurious MOTUs that inflated diversity estimates by a factor of two when compared to true diversity (Marques et al., 2020). As such we provided conservative MOTU diversity estimates where we limited the number of false‐negative MOTUs while also removing many false positives. Pseudo‐genes were unlikely to bias our analyses because nuclear DNA is rare in eDNA samples (Capo et al., 2021; Stat et al., 2017) and is outnumber by a factor of hundreds to thousands by the mitochondrial eDNA of focus here (Robin & Wong, 1988).

### 2.3 MOTU richness

We first compared MOTU local richness with the expected richness of the species pool in the eight sites. For this, we created MOTU presence–absence matrices containing every replicate of each region. We also compiled fish presence–absence matrices from species lists for each of the eight sites from the literature: Scattered Islands (Grande Glorieuse, number of species = 576; Europa Island, *n* = 506; Juan de Nova Island, *n* = 480; Tromelin Island, *n* = 239; personal communication with Terres Australes et Antarctiques Francais; www.taaf.fr), Santa Marta (*n* = 515; SIBM, 2021), Providencia (*n* = 343; Robertson & Van Tassell, 2019), Malpelo (*n* = 257; Robertson & Allen, 2015), and Guadeloupe (*n* = 425; Froese & Pauly, 2000). As exploratory analyses, we examined whether the transect MOTU richness varied among oceanic regions (*n* = 3) by performing a Kruskal–Wallis rank sum test. We also related the MOTU richness per replicate to the site richness (from species lists) using a linear model (but note the different sequencing platforms between regions). We estimated the recovered MOTU richness for each filtration replicate per transect and determined if the mean α‐diversity differed between paired filtration replicates for a given transect using a Wilcoxon signed‐rank test.

### 2.4 MOTU compositional dissimilarity

To understand the variability in MOTUs recovered between filtration replicates, we quantified the compositional similarity of MOTUs. We estimated the pairwise Jaccard's dissimilarity index (*β*_jac_) between filtration replicates per transect using the R package *vegan* (Oksanen et al., 2019). The Jaccard index ranges from 0 (species composition between the replicates is identical, that is, complete similarity) to 1 (no species in common between the replicates, i.e., complete dissimilarity). We partitioned the Jaccard index into turnover (*β*_jtu_) and nestedness (*β*_jne_) components using the R package *betapart* (Baselga & Orme, 2012). Nestedness quantifies the extent to which replicates are subsets of each other. Turnover indicates the amount of species replacement among replicates, that is, the substitution of species in one replicate by different species in the other one (Baselga & Orme, 2012; Legendre & De Cáceres, 2013). In addition, we tested whether *β*_jac_ differed between the regions using a Kruskal–Wallis rank sum test.

### 2.5 Local‐scale MOTU accumulation curves

To analyze the local‐scale richness accumulation, we repeated circular transects multiple times in Malpelo and Santa Marta. We sampled two locations in Santa Marta filtrating 6 replicates at each within 20 hr and one location in Malpelo filtrating 10 replicates within 3 days. This sampling design defined three local MOTU accumulation “experiments.” We produced MOTU richness accumulation curves across filtration replicates from each location using the *specaccum* function from the R package *vegan* (Oksanen et al., 2019). The *“* *random* *”* method was used to generate 1,000 accumulation curves which were used to fit 14 models using the *sar_average* function in the R package *sars* (Matthews et al., 2019; models fitted: ‘powerR’, ‘emp1’, ‘loga’, ‘koba’, ‘mmf’, ‘monod’, ‘negexpo’, ‘chapman’, ‘weibull’, ‘asymp’, ‘ratio’, ‘weibull4’, ‘betap’, ‘heleg’). These models described how the number of replicates predicts MOTU richness based on 14 different mathematical functions. We compared model fits selecting the model with the lowest AIC. We generated multimodel mean averages which were used for asymptote calculations, extrapolation, and visualization. We next used the *sar_pred* function to extrapolate MOTU richness for up to 60 filtration replicates. We defined asymptotes as the number of replicates at which less than 1 new MOTU was added per additional sample.

### 2.6 Regional‐scale MOTU accumulation curves

In contrast to the saturation curves at one location, we assessed the extent to which our eDNA protocol captures regional fish biodiversity. MOTU accumulation curves were calculated using all filtration replicates in each of the eight sites. Species accumulation curves were produced and compared as above (Figure 1; Table 1) rather than within localized repeated transects. All transects and replicates from all stations within a sampling site were pooled to form a site‐wide (or regional) accumulation curve.

All analyses were performed in R version 4.0.1 (R Core Team, 2020). The activities in Malpelo were undertaken with the permit: “Resolución Número 0170‐2018 MD‐DIMAR‐SUBDEMAR‐ALIT 8 de marzo de 2018.”

## 3 RESULTS

### 3.1 Overview of eDNA biodiversity patterns

**FIGURE 2:**
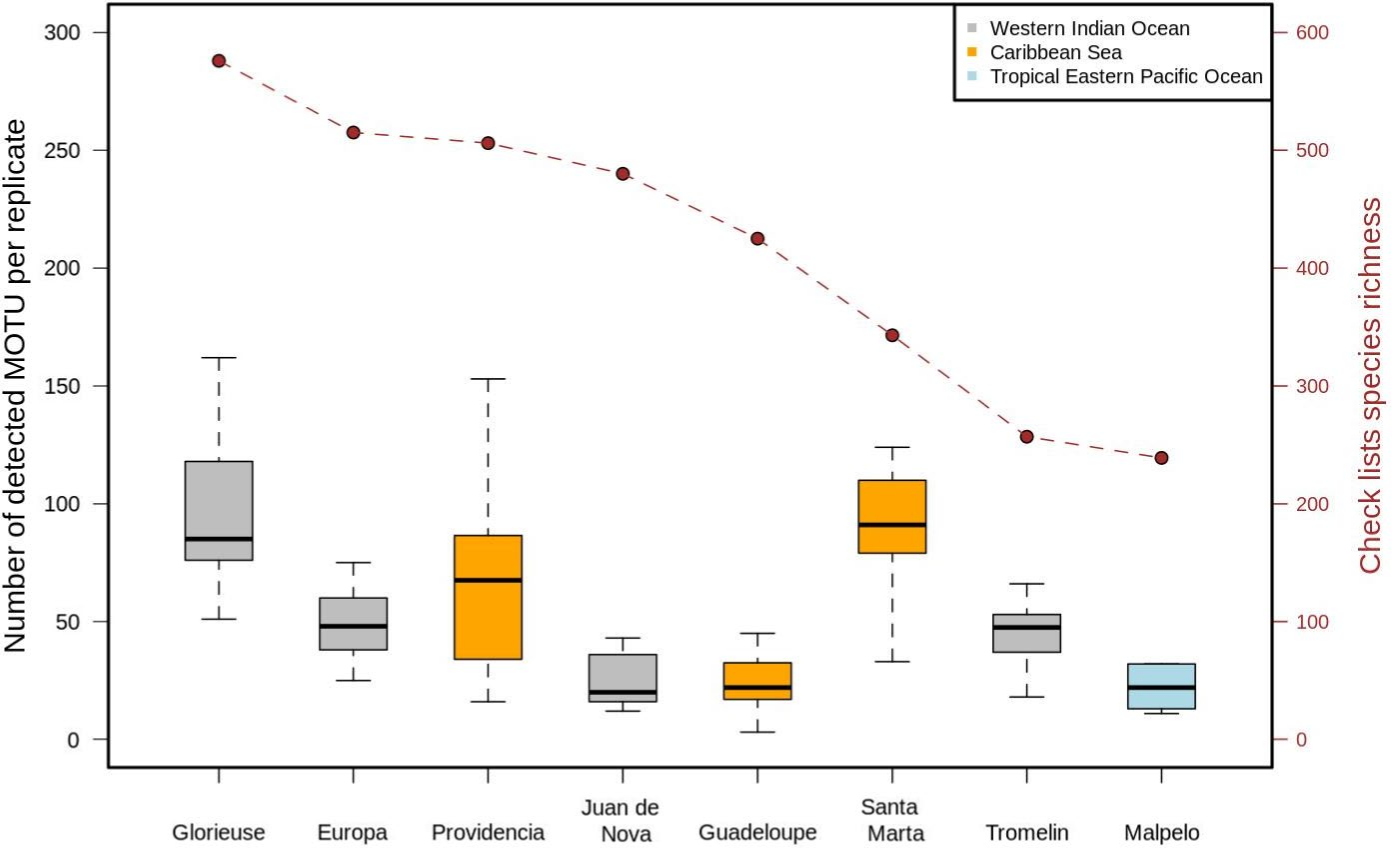
Sites ranked by their expected species richness based on species lists in different regions (see Methods). One single filtration replicate recovered up to 162 fish MOTUs in the most diverse site monitored. Overall, we found an association between the size of the species pool and the number of MOTUs recovered in a single replicate. The relatively consistent proportional MOTU samples from the full species pools suggests that in richer sites filtration replicates were not saturated with available eDNA. The bold central lines correspond to median values across filtration replicates, interquartile range (25th–75th) corresponds to box edges, and whiskers extend to 1.5 × interquartile range

We detected a total of 789 unique MOTUs assigned to bony and cartilaginous fish taxa. Site MOTU richness was significantly and positively associated with the size of the site species pool (slope = 0.1, *t* = 4.7, *p* < .001; Figure 2) reconstructing large‐scale biodiversity gradients across the tropics.

### 3.2 MOTUs richness per replicate

The fish MOTU richness detected by each filtration replicate (*n* = 100) ranged from 3 to 162, with a mean of 58.3 ± 35.6 MOTUs (Figure 2). The mean α‐diversity detected by each filtration replicate for a given transect did not differ significantly (Wilcoxon signed‐rank test: *n* = 50, *Z* = −0.927, *p* = .354). The MOTU richness detected at each transect (i.e., two filtration replicates combined, *n* = 50) ranged from 19 to 184, with a mean of 82.5 ± 42.7 and did not differ significantly among regions (Kruskal–Wallis rank sum test: *χ* ^2^ = 4.0682, *p* = .1308). On average, 69.7% of the MOTU richness along a transect was identified by a single filtration replicate, ranging from 11.5% to 98.1%, with variations among regions (Western Indian Ocean = 63.6%, *n* = 36; Eastern Pacific = 74.5%, *n* = 20; Caribbean = 72.5%, *n* = 44).

### 3.3 MOTU compositional dissimilarity between replicates

**FIGURE 3:**
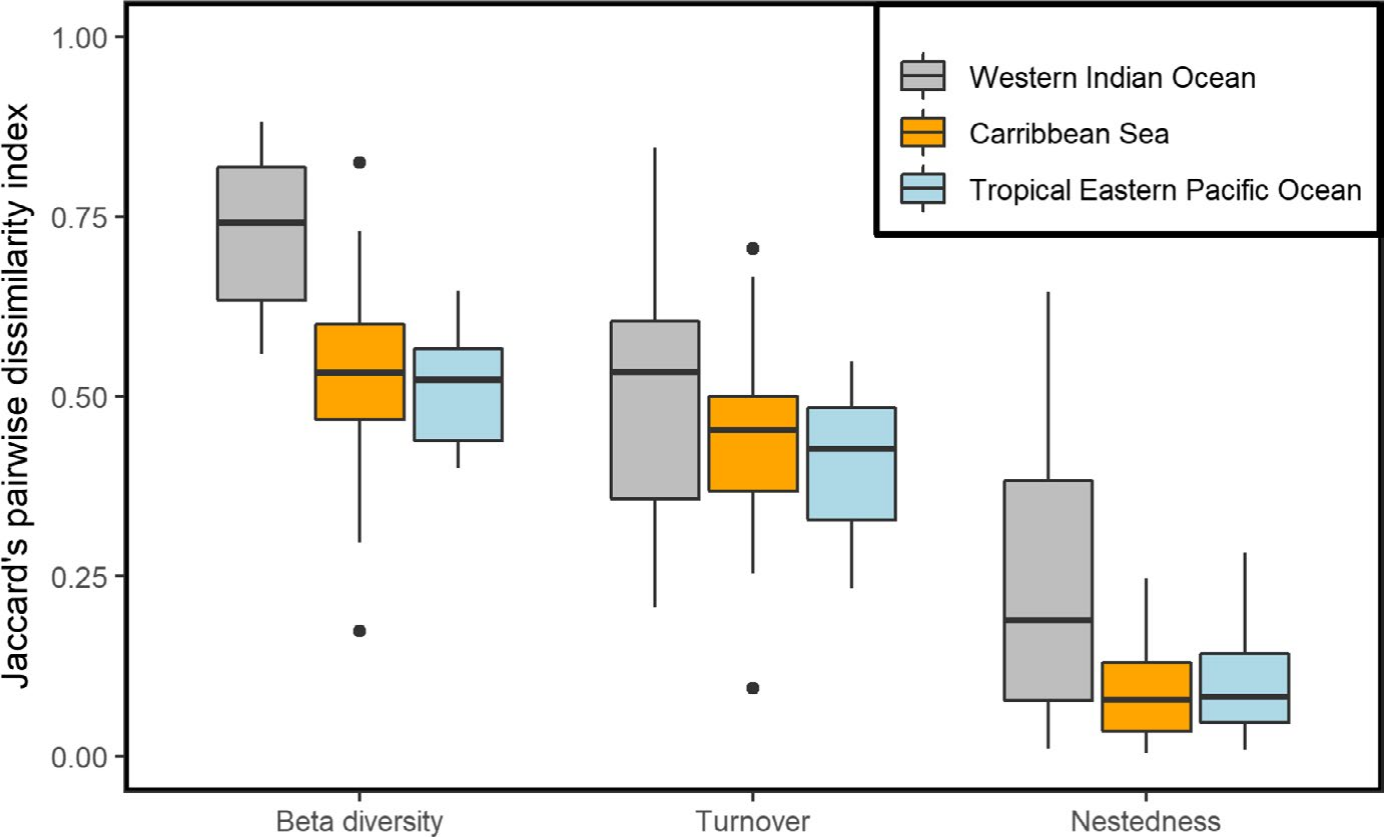
Boxplots of the MOTU compositional dissimilarity between eDNA filtration replicates (***β***‐diversity), partitioned into turnover and nestedness components for three ocean regions. The bold central lines correspond to median values across transects (filtration replicate pairs), interquartile range (25th–75th) corresponds to box edges, and whiskers extend to 1.5 × interquartile range

The composition of fish MOTUs was dissimilar between paired replicates (Figure 3; mean similarity = 0.598 ± 0.155 where 1 is full dissimilarity with no MOTUs in common) and varied among transects ranging from 0.174 to 0.882. The level of dissimilarity between paired replicates varied significantly among regions (Kruskal–Wallis rank sum test: *χ* ^2^ = 22.791; *p* < .001) being most dissimilar in the West Indian Ocean (mean = 0.729 ± 0.102) than in the Caribbean (0.528 ± 0.146) and the Eastern Pacific (0.511 ± 0.081). MOTU compositional differences between replicates were primarily due to MOTU turnover (Figure 3; *n* = 49, *Z* = −6.097, *p* < .001; mean turnover = 0.450 ± 0.153) with a lower contribution of nestedness (mean = 0.149 ± 0.146). Turnover ranged from 0.095 to 0.846 and nestedness from 0.005 to 0.646.

### 3.4 Local‐scale MOTU accumulation curves

**FIGURE 4:**
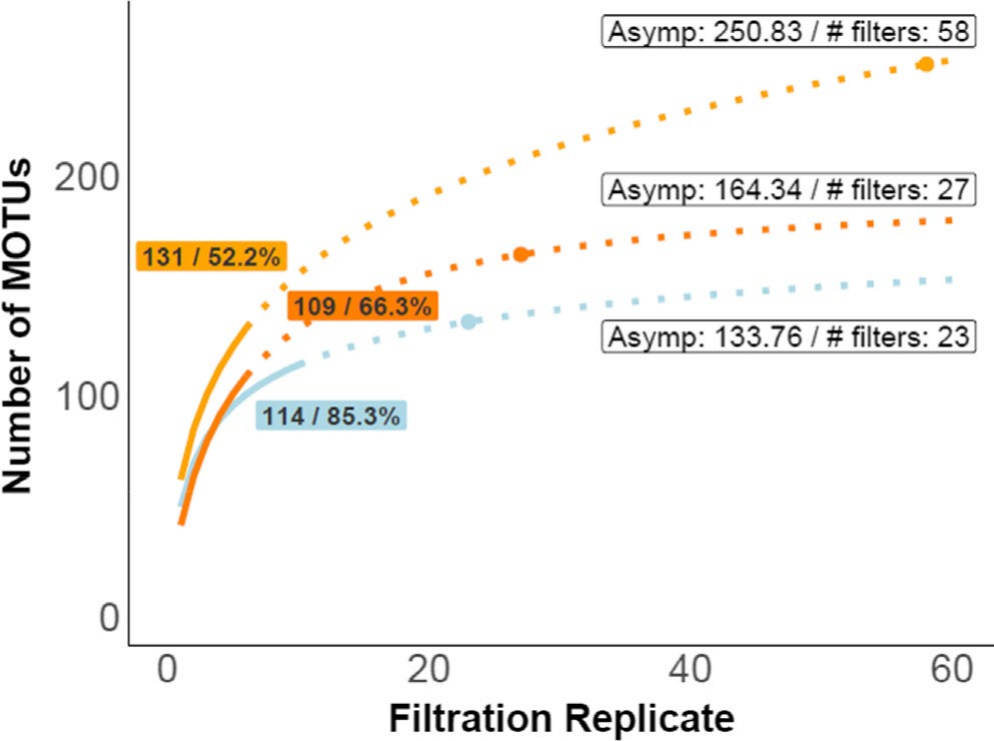
Local‐scale MOTU richness accumulation analysis of eDNA filtration replicates from Santa Marta and Malpelo. The curves show the multimodel mean average of the local MOTU richness and richness extrapolation for the filtration replicates collected by repeated sampling at the same location over a short period. Colored text boxes indicate the final sampled richness and the percentage of the estimated richness asymptote reached with our filtration replicates. Points on the curve mark the asymptote (defined as a < 1 MOTU increase in species richness per added sample). The asymptotic MOTU richness plus the number of filters required to reach the asymptote are noted in the white text box next to the curves. The solid line shows the richness of the filters collected during actual sampling; the dotted line is the extrapolation of richness up to 60 filters. The curve color corresponds to the sampling regions: Santa Marta (light orange: “tayrona_camera_1,” dark orange: “tayrona_camera_2”), Malpelo (blue). See Figure S1 for the same analysis conducted on MOTUs assigned to the nearest taxonomic rank

The accumulated fish MOTU richness in the two locations in Santa Marta was between 109 and 131, and the one location in Malpelo was 114. After 6–10 replicates sampling in the same location, MOTU richness did not fully saturate (defined as <1 additional MOTU per filtration replicate) with additional replicates adding new MOTUs to the total (Figure 4). Modeled accumulation curves suggest that 27–58 filtration replicates would be required to reach an asymptotic richness of 164–251 MOTUs in Santa Marta, and 23 filtration replicates to reach an asymptotic richness of 134 MOTUs in Malpelo.

### 3.5 Regional‐scale MOTU accumulation curves

**FIGURE 5:**
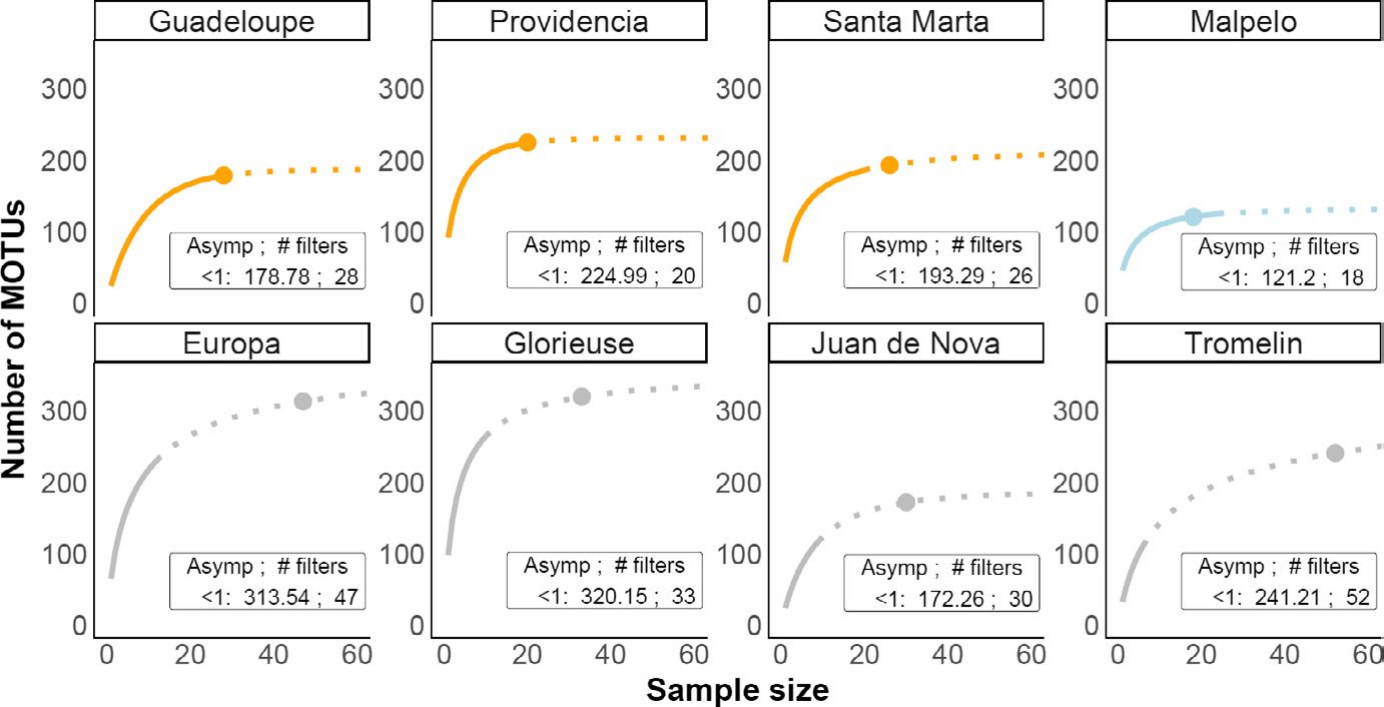
Regional MOTU richness accumulation curves of eDNA filtration replicates across the Caribbean, Eastern Pacific, and Western Indian Ocean. The curves show the multi‐model mean averages of the local richness and richness extrapolation (number of MOTUs) for the number of filters (sample size) from each region. Points on the curve represent the asymptote (defined as a less than 1 MOTU increase in species richness per added sample). The asymptote for the MOTU richness plus the number of filters needed to reach the asymptote is noted in the text box below the curves. The solid line shows the richness of the filters collected; the dotted line is the extrapolation of richness up to 60 filters. The colors of the curves correspond to the sampling area: Caribbean Sea (orange), Eastern Pacific (light blue), and Western Indian Ocean (grey)

At a regional scale, MOTU accumulation curves detected various proportions of the total asymptotic MOTU richness (98.8% on average in the Caribbean Sea, 103.3% on average in Malpelo, 67.4% on average in the Western Indian Ocean). The Caribbean and Eastern Pacific filtration replicates saturated MOTU richness after 18–28 replicates (i.e., within our number of replicates), except for Santa Marta, where an additional 6 replicates are predicted to be required to reach an asymptote (Figure 5). In the Western Indian Ocean, where sampling was less exhaustive, regional MOTU richness did not saturate and reached between 46.4% (Tromelin) and 82.7% (Grande Glorieuse) of the predicted asymptotic MOTU richness. To reach an asymptotic richness of 172.3–320.2 MOTUs in the Western Indian Ocean, our estimates suggest that between 30 and 52 replicates would be required. The shapes of regional accumulation curves were qualitatively different between the three oceans and showed differing levels of both diversity and sampling exhaustiveness across sites (Figure 5). Our results were qualitatively insensitive to the definition of the asymptote used (see Figures S2‐S3).

## 4 DISCUSSION

Our results reveal that without adequate replication, or high water volumes, sampling variability may undermine biodiversity estimates in highly diverse tropical ecosystems, such as coral reefs (Bessey et al., 2020; Cilleros et al., 2019; Juhel et al., 2020; Polanco Fernández et al., 2020). The variability of eDNA biodiversity estimates showed high compositional dissimilarity between filtration replicates so, for some locations, even extensive sampling with 6–20 × 30 L replicates did not reach an asymptotic number of detected MOTUs to provide stable biodiversity estimates. Interestingly, there are little difference in overall MOTU richness between paired replicates, although these replicates recover different species identities so are essential in eDNA sampling design. Promisingly, the regional biodiversity of tropical coral reef systems was reliably quantified through repeated eDNA sampling which suggests that well‐designed eDNA studies (large volume, multiple replicates) could provide robust broad‐scale biodiversity estimates.

The similar MOTU richness but different composition between replicates suggests that eDNA distribution varies at a fine scale in seawater and is certainly patchier than previously thought. This patchiness of fish eDNA in tropical reefs is further supported by Bessey et al. (2020) who report that multiple collections from a single site 2m apart have <30% overlapping species detections. The fine‐scale distribution of eDNA in the environment could be a function of multiple factors. For example, ambient eDNA in seawater could be modified by complex sea currents, surface slicks (Whitney et al., 2021), local water dynamics, thermohaline circulation forces, spatiotemporal variation in organism activity, and behaviors (e.g., spawning, feeding, diel migrations), different DNA shedding, degradation, and decay rates (Harrison et al., 2019). Identifying when, where, and to what extent these varying processes act to modify spatial and temporal eDNA distribution is critical to disentangle biodiversity variation from sampling variation on reefs.

Since biodiversity changes are most often detected as compositional turnover, but not necessarily richness changes, we highlight a major challenge in developing eDNA to monitor ecosystem modifications through space and time (Blowes et al., 2019; Dornelas et al., 2014; Hill et al., 2016; Santini et al., 2017). Our results imply that if sample variability is not accounted for, or survey designs are not well replicated, eDNA‐derived time series could over‐emphasize compositional turnover by containing many false negatives. This point will be exacerbated where incomplete reference databases recover a small portion of common species and falsely identify low species turnover among samples (Schenekar et al., 2020), even though MOTU turnover identified here may be very high. We found MOTU compositional differences between replicates to be higher in the more speciose Western Indian Ocean (under similar sampling protocols), perhaps due to the larger species pool, further challenging eDNA applications in most diverse tropical systems (Juhel et al., 2020). Current protocols should be cautiously applied to biomonitoring if such limitations remain unresolved. Our results also imply that many replicates of >30 L water are needed to reach a stable estimate of total local biodiversity. Promisingly, the regional biodiversity of tropical systems was relatively well‐quantified through repeated eDNA sampling (e.g., Figure 5), and more exhaustive biodiversity estimates may be achieved by including mesophotic coral ecosystems (from −300 m depth to subsurface) and various habitats (e.g., lagoons, reef‐slope, mangroves, seagrass) (Juhel et al., 2020).

The well‐established community pattern that many species are rare and few are common (McGill et al., 2007) also likely exists in eDNA particles. Moreover, finding rare eDNA fragments in any given sample may be exacerbated by features of marine systems. For example, we likely sampled vagrant open‐ocean species that pass through temporarily, in some of our remote sites (e.g., Malpelo) which may have increased sampling variability. Compared to terrestrial systems, the seawater environment may homogenize eDNA that comes from different habitats (e.g., coral, rock, sand, seagrass). The eDNA species pool could be larger in a seawater sample than expected based on habitat variation along a given 2 km transect. Dispersion of eDNA between distinct habitats (e.g., from seagrass beds to coral reefs) would enhance the likelihood of finding a rare habitat specialist from a different habitat type and increasing perceived sampling variability. As such, eDNA variability may be greater in seascapes with a greater diversity of habitats. Sampling designs may need to account for the extent that a given water body accumulates sources of eDNA, and the amount of habitat variation that a water sample signal is aggregated over. To use eDNA‐derived data most effectively, statistical analyses may need to control for habitat variations before reaching conclusions (Boulanger et al., 2021).

Marine eDNA protocols are challenged by the compositional turnover between replicates. As in traditional approaches, saturation of biodiversity samples only occurs with many replicates on tropical reefs (MacNeil et al., 2008). However, traditional methods like underwater visual census (UVC) and baited remote underwater video (BRUVs) are systematically biased by observer effects and fish behavior, leading to false negatives for cryptic and elusive species (Ackerman & Bellwood, 2000; Bernard et al., 2013; MacNeil et al., 2008). For example, we found ~30 Chondrichthyes species that typically would not be encountered on visual surveys (e.g., 2 *Mobula* sp., 6 *Carcharhinus* sp.; Polanco Fernández et al., 2020), among other elusive and endangered megafauna that have been uncovered during similar sampling regimes (Juhel et al., 2021). We highlight that eDNA replicates may be affected by factors that contribute to the precision of biodiversity estimates, rather than a systematically biased biodiversity signal as obtained with UVC or BRUVs. For example, a lack of eDNA in water samples leading to availability errors, although see Stat et al. (2019) for biases against specific genera and Kelly et al. (2019) for discussions of primer efficiency biases.

Coral reefs are extremely speciose (Edgar et al., 2017; Fisher et al., 2015) and so 60 L (two replicates) or even 180 L (six replicates) does not seem to fully quantify local biodiversity. Instead, in support of other eDNA studies that filtered far less water, our replicates only sampled a portion of diversity (Bessey et al., 2020; DiBattista et al., 2017; Jeunen, Knapp, Spencer, Taylor, et al., 2019; Juhel et al., 2020; Koziol et al., 2019; Sigsgaard et al., 2019; Stat et al., 2019). In temperate systems, 20 L of water was sufficient for fish family richness to saturate (Koziol et al., 2019; but see Evans et al., 2017), but tropical systems are more challenging to monitor. The number of eDNA replicates to ensure tropical fish diversity saturation varies widely. For example, 32–39 samples of 0.5 L of water began to saturate fish genera diversity in western Australia (Stat et al., 2019), but 92 samples of 2 L did not saturate diversity in West Papua, Indonesia, a hotspot of fish diversity (Juhel et al., 2020). Furthermore, even the largest sample of 2 L in Bessey et al. (2020) only detected <43% (75/176) of the total species pool reported in the Timor Sea.

eDNA accumulation curves often confound site‐accumulated (regional) and replicate‐accumulated (local) diversity presenting challenges for replicate number and water volume refinements (but see Bessey et al., 2020). Comparing available estimates, integrative sampling (performed here), rather than point sampling, for example, Stat et al. (2019) and Juhel et al. (2020), appears very promising. For example, in Caribbean and Eastern Pacific sites within ~25 filters, we found additional filters added only <1 MOTU. Previous works using point samples have far higher sampling numbers, and higher DNA analysis costs per filter so leading to apparently lower cost‐effectiveness (unless filters are aggregated at the DNA extraction step; e.g., Juhel et al., 2020; Stat et al., 2019). Future work should optimize sampling designs and the trade‐off between water sample volume and replicate number, which we only partially explore, and how these factors contribute to the precision of biodiversity estimates in controlled settings (Miya et al., 2015). For example, if sampling nearer to substrate bottoms greatly improves recovery of eDNA this additional cost (e.g., divers, submersibles, and additional expertise) could work out as a cost‐effective solution to address surface sampling variability. Another option would be to use previous knowledge of biodiversity in each site to adapt the number of replicates to reach expected saturation.

A similar pattern of low compositional similarity, and consistent richness in replicates, could arise if filters saturate with eDNA and prevent the full quantification of biodiversity. Our analyses suggest this is unlikely because the richness recovered from the eDNA filters was associated with the size of the species pools, which would be unexpected if filters had a maximum richness capacity that was reached consistently. Furthermore, we might expect nestedness to be more important if filters or PCR processes were first saturated with the most commonly available eDNA, but we found MOTU compositional differences between replicates were more strongly related to turnover than nestedness. Finally, if filters first saturate with common species, eDNA recovery of rare species would be limited, but in our eDNA protocol, we find many species that remain undetected or rare in visual surveys (Polanco Fernández et al., 2020). Promisingly, this suggests not only that our sampling protocol is robust but also that sampling and filtering an even greater water volume per filtration replicate is a feasible approach to better quantify the high fish diversity of coral reefs. Given the low biomass‐to‐water ratio in marine systems, a high volume of filtered water is likely a prerequisite to have a representative sampling of the marine environment (Bessey et al., 2020). However, other parameters must be considered and explored in the future to identify whether physicochemical and local oceanographic conditions introduce variability in biodiversity estimates (Collins et al., 2018).

## 5 CONCLUSION

Our findings underline both promises and limitations of eDNA derived biodiversity estimates in hyperdiverse tropical ecosystems. On one hand, local richness estimation appears to rapidly resolve broad‐scale richness patterns of underdocumented tropical marine biodiversity (Costello et al., 2010; Menegotto & Rangel, 2018). On the other hand, stochasticity between sample replicates urges cautious application to biomonitoring, and further protocol refinement, to avoid misattribution of biodiversity trends to detection errors. A better understanding of the behavior of eDNA in diverse physicochemical marine environments will help design more effective eDNA sampling protocols and disentangle sampling errors from true biodiversity patterns (Harrison et al., 2019). Resolving whether more replicates, or greater water volumes, leads to higher probability of eDNA recovery is critical for cost‐effective eDNA protocols—but integrative sampling of tens of liters along boat transects appears a promising approach. Using multiple primer sets may also improve the rate of biodiversity sampling saturation but this possibility remains unexplored here. We also recommend testing various water sampling strategies, for example sampling not only surface water, but taking eDNA along a depth gradient where the ecology of eDNA may differ. Accurate, cheap, and fast biodiversity estimates are critically needed to monitor changes in the Anthropic Ocean. Current eDNA protocols provide higher and more realistic estimates of biodiversity than traditional methods for a given sampling effort. This opens very promising and realistic perspectives to quantify biodiversity since increasing the volume of water filtered and replicate numbers is feasible, particularly in regions with high biodiversity. Further refinement of our marine eDNA protocol will better quantify, monitor, and manage changing tropical marine biodiversity.

## CONFLICT OF INTEREST

None declared.

## AUTHOR CONTRIBUTIONS

**Salomé Stauffer:** Conceptualization (equal); data curation (equal); formal analysis (equal); investigation (equal); methodology (equal); software (equal); validation (equal); visualization (equal); writing–original draft (equal); writing–review and editing (equal). **Meret Jucker:** Conceptualization (equal); data curation (equal); formal analysis (equal); investigation (equal); methodology (equal); software (equal); validation (equal); visualization (equal); writing–original draft (equal); writing–review and editing (equal). **Thomas Keggin:** Conceptualization (equal); investigation (equal); supervision (equal); writing–review and editing (equal). **Virginie Marques:** Data curation (equal); formal analysis (equal); investigation (equal); software (equal); writing–review and editing (equal). **Marco Andrello:** Investigation (equal); resources (equal); writing–review and editing (equal). **Sandra Bessudo:** Funding acquisition (equal); investigation (equal); project administration (equal); resources (equal); writing–review and editing (equal). **Marie‐Charlotte Cheutin:** Investigation; writing–review and editing. **Giomar Helena Borrero‐Pérez:** Investigation (equal); Methodology (equal); writing–review and editing (equal). **Eilísh Richards:** Investigation (equal); supervision (equal); writing–review and editing (equal). **Tony Dejean:** Conceptualization; data curation (equal); formal analysis (equal); supervision (equal); writing–review and editing (equal). **Régis Hocdé:** Funding acquisition (equal); investigation (equal); methodology (equal); project administration (equal); supervision (equal); writing–review and editing (equal). **Jean‐Baptiste Juhel:** Conceptualization; investigation (equal); project administration (equal); supervision (equal); writing–review and editing (equal). **Felipe Ladino:** Project administration (equal); resources; writing–review and editing (equal). **Tom B. Letessier:** Investigation (equal); writing–review and editing (equal). **Nicolas Loiseau:** Investigation (equal); writing–review and editing (equal). **Eva Maire:** Investigation (equal); project administration (equal); supervision (equal); writing–review and editing (equal). **Maria Mutis Martinezguerra:** Investigation (equal); methodology (equal); writing–review and editing (equal). **Stéphanie Manel:** Data curation (equal); formal analysis (equal); funding acquisition (equal); supervision (equal); writing–review and editing (equal). **David Mouillot:** Funding acquisition (equal); supervision (equal); writing–review and editing (equal). **Andrea Polanco Fernández:** Investigation (equal); methodology (equal); writing–review and editing (equal). **Alice Valentini:** Data curation (equal); formal analysis (equal); methodology (equal); validation (equal); writing–review and editing (equal). **Laure Velez:** Investigation (equal); writing–review and editing (equal). **Camille Albouy:** Formal analysis (equal); investigation (equal); project administration (equal); writing–review and editing (equal). **Loïc Pellissier:** Conceptualization; investigation (equal); methodology (equal); supervision (equal); validation (equal); writing–original draft (equal); writing–review and editing (equal). **Conor Waldock:** Conceptualization (equal); investigation (equal); supervision (equal); validation (equal); visualization (equal); writing–original draft (equal); writing–review and editing (equal).

## Supporting information

Fig S1‐3

## Data Availability

We agree to archive our data in Dryad on acceptance of our manuscript.
