# Supplementary material for: How many replicates to accurately estimate fish biodiversity using environmental DNA on coral reefs?
Source: Ecol Evol. 2021 Oct 6;11(21):14630–43. doi: 10.1002/ece3.8150 (PMC8571620; doi:10.1002/ece3.8150)
Supplement: Supplementary file 1 — Fig S1‐3 [file ECE3-11-14630-s001.docx]

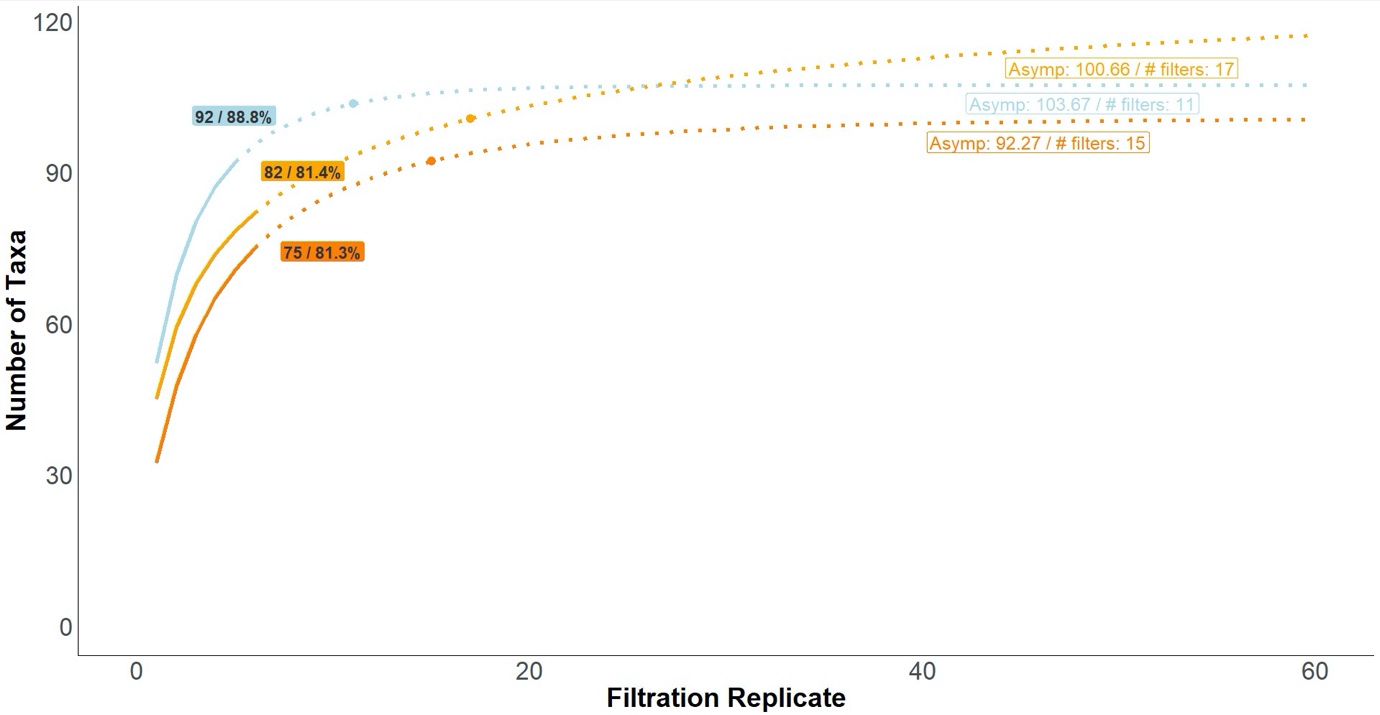


**Figure S1.** Local-scale taxonomic richness accumulation analysis of eDNA filtration replicates from Santa Marta and Malpelo. Taxonomic assignment is determined based on sequence similarity of MOTUs to NCBI reference database using ECOTAG software (see Polanco-Fernandez et al. 2020 for details). The curves show the multi-model mean average of the local taxonomic richness and richness extrapolation for the filtration replicates collected by repeated sampling at the same location over a short period. Coloured text boxes indicate the final sampled richness and the percentage of the estimated richness asymptote reached with our filtration replicates. Points on the curve mark the asymptote (defined as a < 1 taxa increase in species richness per added sample). The asymptotic taxonomic richness plus the number of filters required to reach the asymptote are noted in the white text box next to the curves. The solid line shows the richness of the filters collected during actual sampling; the dotted line is the extrapolation of richness up to 60 filters. The curve colour corresponds to the sampling regions: Santa Marta (light orange: ‘tayrona_camera_1’, dark orange: ‘tayrona_camera_2’), Malpelo (blue).


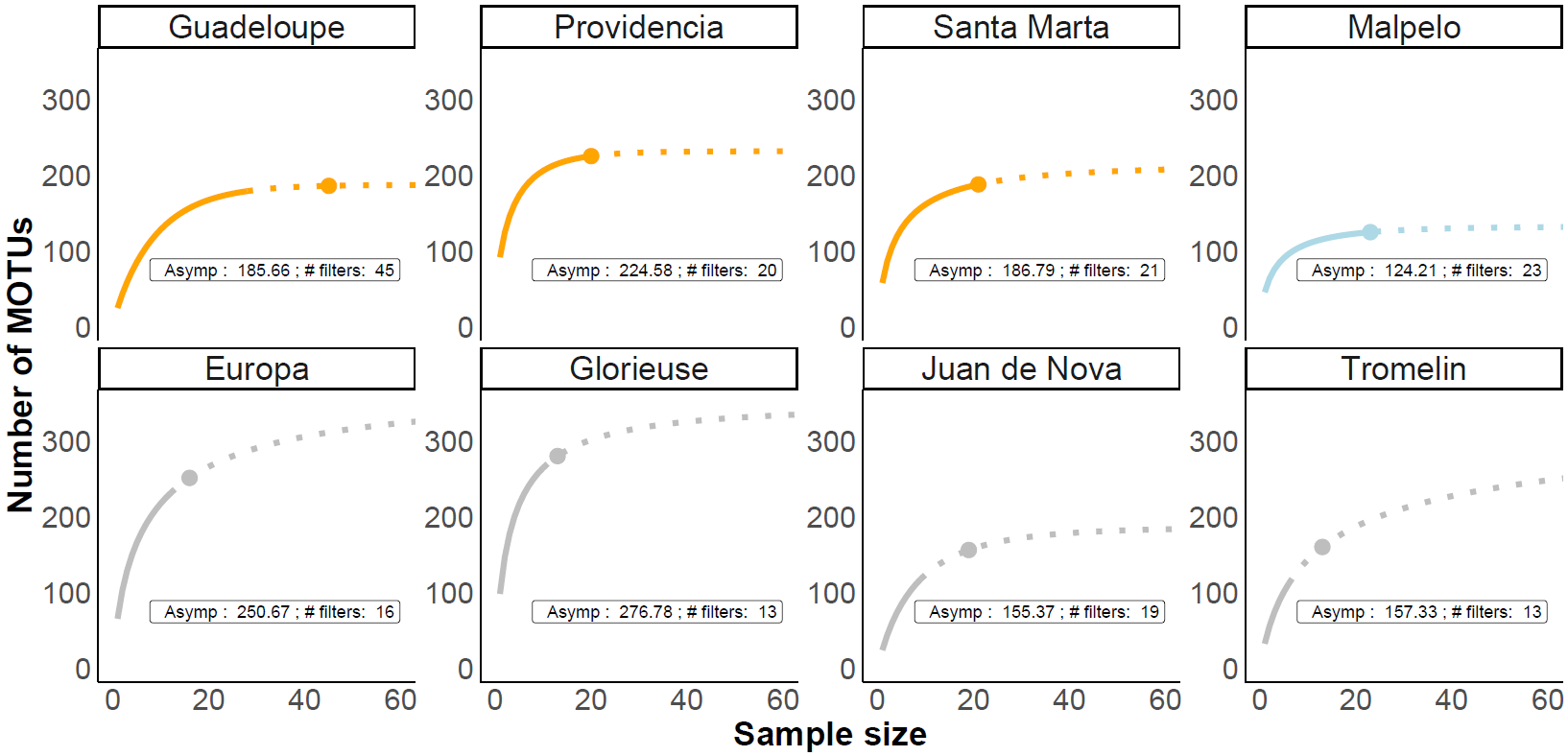


**Figure S2.** Regional MOTU richness accumulation curves of eDNA samples across the Caribbean, Eastern Pacific and Western Indian Ocean. The curves show the multi-model overlap of the local richness and richness extrapolation (number of MOTUs) for the number of filters (sample size) from each region. Points on the curve represent the asymptote (given by the asymptote model output from the SARS package). The asymptote for the MOTU richness plus the number of filters needed to reach the asymptote are noted in the text box below the curves. The solid line shows the richness of the filters collected; the dotted line is the extrapolation of richness up to 60 filters. The colours of the curves correspond to the sampling area: Caribbean Sea (orange), Eastern Pacific (light blue), Western Indian Ocean (grey).


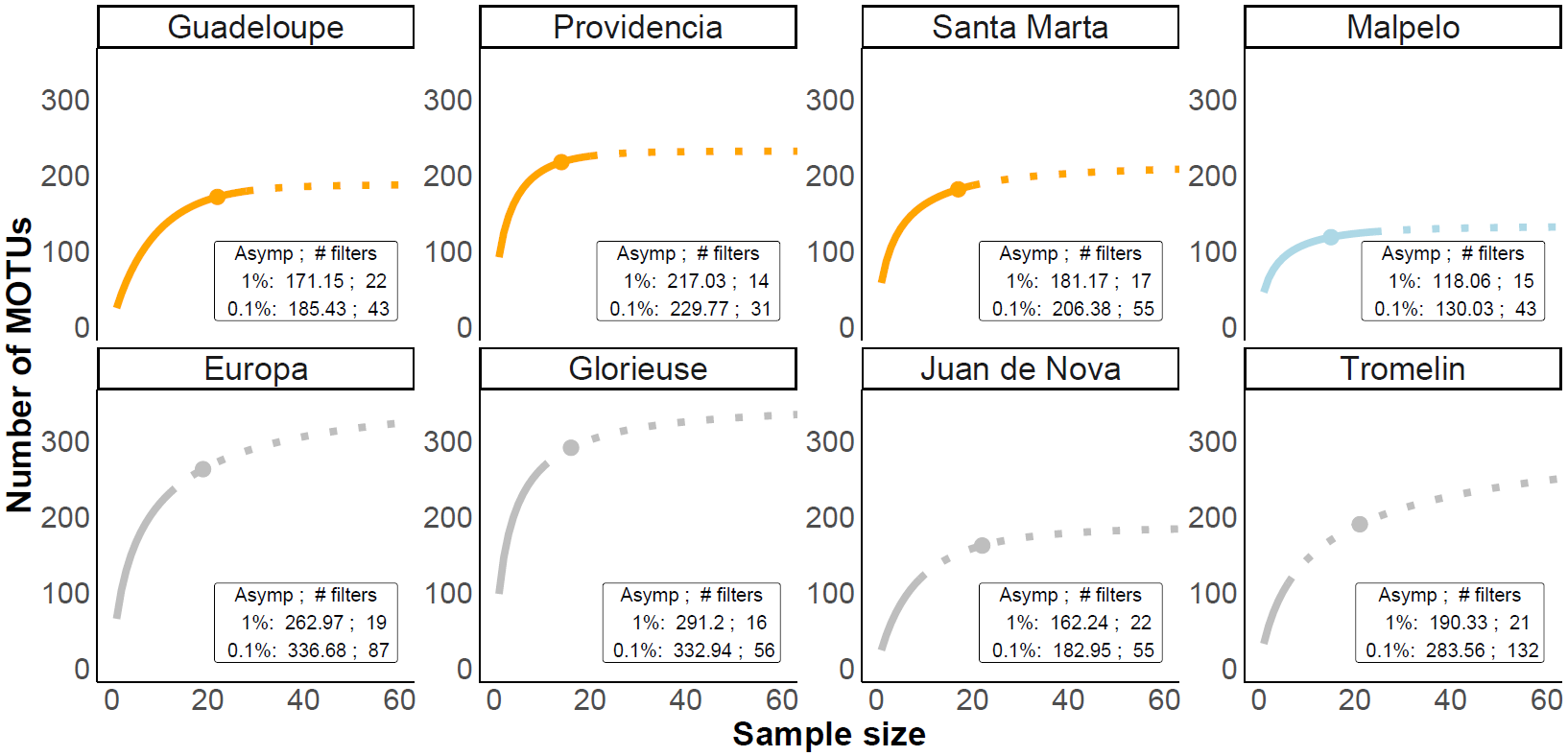


**Figure S3.** Regional MOTU richness accumulation curves of eDNA samples across the Caribbean, Eastern Pacific and Western Indian Ocean: The curves show the multi-model overlap of the local richness and richness extrapolation (number of MOTUs) for the number of filters (sample size) from each region. Points on the curve represent the asymptote (defined as a less than 1% increase in species richness per added sample). The 1% and 0.1% asymptote for the MOTU richness plus the number of filters needed to reach the asymptote are noted in the text box below the curves. The solid line shows the richness of the filters collected; the dotted line is the extrapolation of richness up to 60 filters. The colours of the curves correspond to the sampling area: Caribbean Sea (orange), Eastern Pacific (light blue), Western Indian Ocean (grey).
